# Hippocampal phospho-tau/MAPT neuropathology in the fornix in Alzheimer disease: an immunohistochemical autopsy study

**DOI:** 10.1186/s40478-016-0388-2

**Published:** 2016-10-28

**Authors:** Edward D. Plowey, Jennifer L. Ziskin

**Affiliations:** Department of Pathology, Stanford University School of Medicine, Edwards Building, Room R-241, 300 Pasteur Drive, Stanford, CA 94305 USA

**Keywords:** Tau/MAPT, Hippocampus, Fornix, Basal forebrain, Alzheimer disease

## Abstract

Whereas early Alzheimer disease (AD) neuropathology and mild cognitive impairment are relatively common in aging, accurate prediction of patients that will progress to dementia requires new biomarkers. Recently, substantial work has focused on phospho-tau/MAPT (p-MAPT) neuropathology since its regional propagation correlates with the degree of cognitive impairment in AD. Recent diffusion tensor imaging studies in AD suggest that increased diffusion in the fornix secondary to p-MAPT-related axonal injury could serve as a predictive biomarker of the risk of disease progression. However, our knowledge of p-MAPT neuropathology in the fornix is limited. To address this gap in knowledge, we examined p-MAPT neuropathology in the fornix and basal forebrain nuclei via AT8 immunohistochemistry in 39 brain autopsies spanning the spectrum of AD neuropathologic changes. We found that the fornix and its precommissural efferent target nuclei (septum and nucleus accumbens) demonstrated neuronal and thread-like p-MAPT neuropathology only in National Institute on Aging/Alzheimer Association (NIA/AA) stages B2 and B3 of neurofibrillary degeneration, consistent with involvement after (and propagation from) the hippocampal formation. Interestingly, although tau astrogliopathy was frequently observed in the mammillary bodies in stage B2, neuronal tauopathy was not observed in the postcommissural targets (mammillary bodies and anterior thalamic nucleus) until stage B3. Tauopathy in the nucleus basalis of Meynert was strongly correlated with p-MAPT-positive axons in the fornix, suggesting that projections to the hippocampus also likely contribute to fornix tauopathy. Our cross-sectional autopsy findings indicate that the fornix is involved by p-MAPT neuropathology secondary to hippocampal involvement by AD neuropathology. Furthermore, our findings are compatible with the goal of *in vivo* detection of p-MAPT-related axonal pathology in the fornix in AD as a possible biomarker of p-MAPT progression from the hippocampal formation and underscore a need for additional clinical-radiologic-pathologic correlation studies.

## Introduction

Alzheimer disease (AD) is a common age-related neurodegenerative disease that is typically characterized by an early stage of amnestic mild cognitive impairment which can progress to multi-domain cognitive deterioration and dementia. Recent decades of clinical studies and bench research have rendered critical insights into the pathologic mechanisms of the disease, but efforts to translate mechanistic insights into effective disease treatments have yet to achieve therapeutic goals. One potential explanation for the inefficacy of new experimental treatments is that they have been studied in patients with advanced disease when a window for functional recovery has closed. This possibility mandates a better understanding of early AD neuropathology as well as the discovery of novel biomarkers that herald disease onset and that accurately identify the risk for progression from mild cognitive impairment (MCI) to dementia in sporadic AD.

AD is defined neuropathologically by dual proteinopathies: 1. extracellular plaques comprised of amyloid-beta (Aβ) and 2. intracellular neurofibrillary tangles comprised of phosphorylated microtubule associated protein tau (p-MAPT). Evidence suggests that both neuritic plaques (a subset of extracellular Aβ plaques demonstrating argyrophillic, p-MAPT-immunoreactive dystrophic neurites) and neurofibrillary tangles contribute to cognitive impairment [15–17, 21], however their relative contributions and interactions are not completely understood. The National Institute on Aging/Alzheimer Association (NIA/AA) AD staging system [12], which is based in part on the Braak staging system of neurofibrillary degeneration [2–4], delineates the typical progression of cerebral neurofibrillary degeneration in AD beginning in the transentorhinal and entorhinal cortices (B1), progression into the hippocampal formation (B2) and then culminating in involvement of the association neocortices followed by the primary neocortices (B3). This stereotypical progression suggests a possible role for axons and synapses in the p-MAPT neuropathology propagation. The importance of p-MAPT neuropathology is underscored by the strong correlation between neocortical neurofibrillary degeneration and cognitive impairment in AD demonstrated in numerous clinicopathologic correlation studies (reviewed in Nelson et al. 2012 [16]). Whereas entorhinal NFTs are a near universal finding in cognitively intact elderly adults, amnestic MCI is associated with limbic stage neurofibrillary degeneration and multi-domain cognitive impairment in AD is highly correlated with isocortical neurofibrillary tangles [16]. Although there is compelling evidence that a small but significant proportion of AD presents with atypical clinical features and progression of neurofibrillary degeneration, especially in young patients [13], the typical progression of tau neuropathology suggests that imaging modalities to detect tau spread *in vivo* may render novel AD biomarkers. Furthermore, diffusion tensor imaging (DTI) has recently received attention as a modality to detect p-MAPT-related axonal injury in the fornix in AD [1, 18]. These advances have a great potential to reveal novel insights into biomarkers that can predict patients at risk of progression from MCI to dementia in AD.

Our search of the literature suggested that the propagation of p-MAPT neuropathology from the hippocampus in AD is incompletely understood. To our knowledge, p-MAPT neuropathology in the fornix in the spectrum of AD neuropathologic changes has not been reported. The fornix is a major tract of efferent fibers from the hippocampal formation (reviewed in Duvernoy et al. [9]). Efferent fibers of the postcommissural fornix innervate the mammillary body and anterior thalamic nucleus in the regulation of memory. Efferent fibers of the precommissural fornix innervate the septum in the regulation of memory and the nucleus accumbens in the control of movement. The fornix also carries afferent fibers into the hippocampal formation, notably cholinergic fibers from the septum and nucleus basalis of Meynert in the control of memory. Considering its important role in hippocampal memory regulation, it is important to know if the fornix if involved by p-MAPT neuropathology in AD and whether fornix-related imaging biomarkers might detect sequelae of p-MAPT related injury versus non-specific changes.

In this study, we sampled and performed p-MAPT immunohistochemistry on the fornix and efferent and afferent structures of the hippocampal formation in 39 recently archived brain autopsies. The goal was to determine whether, and at which NIA/AA stage of neurofibrillary degeneration, the fornix is involved by p-MAPT neuropathology in AD. Our cross-sectional data confirm p-MAPT neuropathology in the fornix when AD-related neurofibrillary degeneration affects the hippocampal formation. Furthermore, our results are compatible with sequential anterograde p-MAPT neuropathology propagation from the hippocampal formation to the basal forebrain nuclei via the fornix in early AD but also suggest the possibility of propagation of p-MAPT neuropathology into the hippocampal formation via the fornix from the basal forebrain. Our results are also compatible with the possibility that modalities to detect p-MAPT neuropathology propagation or p-MAPT-related axonal injury/loss in the fornix could potentially provide the basis for novel AD biomarkers.

## Materials and methods

### Case selection and tissue harvesting

Adult brain autopsies (118 in total) at Stanford University School of Medicine from April, 2012 through March, 2014 were examined in this study. To mitigate the risk of false negative p-MAPT and Aβ staining due to antigen loss with chronic formalin exposure, tissue from cases no older than 2 years were stained and analyzed. Archived hematoxylin and eosin (H&E) stained autopsy slides were first screened for exclusionary criteria including global hypoxic-ischemic encephalopathy, cerebral edema, temporal lobe infarcts, demyelination or hemorrhage, primary or metastatic brain neoplasms, clinical history of seizures, hippocampal sclerosis, prion disease, chronic traumatic encephalopathy, frontotemporal lobar degeneration, transitional or diffuse Lewy body disease, diffuse cerebral infections and hemosiderosis. Archived H&E stained slides from the remaining brain autopsies of patients ≥ 65 years of age were next screened for hippocampal neurofibrillary tangles on hematoxylin and eosin (H&E)-stained sections. All cases with hippocampal neurofibrillary tangles evident on H&E and a random subset of cases without hippocampal neurofibrillary tangles evident on H&E were selected for tissue sampling of hippocampal formation efferent structures. The data include 11 cases from 2011-early 2012, which were analyzed as a pilot cohort in 2013, and an AD case from mid-2015.

We next examined the archived autopsy brains and harvested the following tissue sections for analysis of p-MAPT immunostaining: anterior hippocampus at the level of the mammillary body; middle frontal and superior temporal gyri at the level of the mammillary body; mammillary bodies (bilateral); intraventricular septum; nucleus accumbens; anterior commissure and nucleus basalis of Meynert; fornix (coronal sections of the body); anterior thalamic nucleus. For the body of the fornix, 2–3 coronal sections from each case were harvested. Tissue blocks were processed for histology and embedded in paraffin using routine methods. Tissue sections (6 μm thickness) were stained with H&E to confirm that the sampled structures were indeed represented. In addition to the above sections, additional tissue blocks from the diagnostic autopsy evaluations containing the middle frontal gyrus, caudate, putamen, midbrain and cerebellum were also utilized for the detection of plaques.

### Special stains

Sections of the harvested structures underwent antigen retrieval with citrate buffer, were blocked with Power Block (Biogenex) and stained via immunoperoxidase reactions using diaminobenzidine chromogenic reactions (Biogenex). The following primary antibodies were used in the immunoperoxidase reactions: phospho-MAPT (clone AT8; Thermo Scientific, MN1020, 1:2,000) and amyloid-beta (clone 4G8, BioLegend, SIG-39220, 1:500). All immunostains for protein aggregates were run alongside appropriate positive control cases. Immunoperoxidase stained slides were counterstained with hematoxylin. For the modified Bielschowsky stain, slides were deparaffinized, hydrated and incubated in 20 % silver nitrate for 30 min in a 37° oven. Following rinses in distilled water, slides were incubated in silver nitrate solution with ammonium hydroxide for 30 min at 37°, rinsed and incubated in silver nitrate solution with 1–2 drops of developer solution until the sections turned black with a golden-brown background. After several rinses the slides were incubated in 2 % sodium thiosulfate, rinsed, dehydrated and coverslipped.

All cases were staged for neurofibrillary degeneration according to the Braak staging system using p-MAPT immunoperoxidase stains of the anterior hippocampus at the level of the mammillary body, the middle and superior temporal gyrus at the level of the mammillary body and the striate and parastriate cortices [2]. Argyrophillic grain disease was excluded in all cases on the basis of absence of argyrophillic grains and coiled oligodendroglial bodies on AT8 immunostains as well as Gallyas silver stains performed on select cases. Cases were staged for Aβ neuropathology according to the method of Thal and colleagues [22] in sections of the middle frontal gyrus, hippocampus, caudate, putamen, nucleus basalis of Meynert, midbrain and cerebellar cortex. Neocortical neuritic plaques were scored semi-quantitatively in sections of the middle temporal gyrus stained via the modified Bielschowsky method to determine the Consortium to Establish a Registry for Alzheimer's Disease (CERAD) neuritic plaque score [11, 12].

### Image acquisition and data analysis

Images of p-MAPT staining were acquired with an Olympus BX51 microscope outfitted with an Olympus DP73 camera connected to a PC running cellSens Entry v1.11. White-balanced images of the fields with the greatest p-MAPT staining were acquired at the following magnifications: mammillary body (100x); nucleus basalis of Meynert (100x); nucleus accumbens (100x); Septum (100x); Alveus (200x); Anterior thalamic nucleus (200x); Fornix (400x). Images were imported into NIH ImageJ. The blue channel was extracted and thresholded at the following cutoff for each structure: fornix 120, septum 120, nucleus accumbens 145, nucleus basalis of Meynert 120, anterior thalamic nucleus 120. A slightly higher threshold was required in the nucleus accumbens to filter out nuclear hematoxylin staining in some of the oligodendroglial cells. Suprathreshold particles were quantified via the analyze particles function of NIH ImageJ and events less than 9 square pixels in area were filtered out of the analysis. Individual p-MAPT stained events and the total area of suprathreshold pixels as a fraction of the total image area of 1,918,400 pixels. Neurons and astrocytes with suprathreshold AT8 staining were counted manually in the images. Phospho-MAPT immunoreactivity data in all structures were compared between NIA/AA stages B0/B1, B2 and B3 of neurofibrillary degeneration using single factor ANOVAs. Post-hoc group comparisons were performed with Bonferroni tests. Pearson correlation coefficients and Bonferroni probabilities accounting for multiple comparisons were used to correlate basal forebrain tauopathy with Braak stage of neurofibrillary degeneration, basal forebrain with AT8-immunoreactive axons in the fornix and the levels of mammillary body tau astrogliopathy with patient age. All statistical analyses were performed with the SYSTAT v.13 statistical software suite.

## Results

A total of 39 cases were included in this study that satisfied the selection criteria outlined in the Methods. Demographic and neuropathologic data for these 39 cases are presented in Table 1. The cohort was comprised of 3 cases with NIA/AA stage B0, 19 with NIA/AA stage B1, 9 NIA/AA stage B2 and 8 NIA/AA stage B3 stages of neurofibrillary degeneration according to AT8-immunostained sections of 1. the anterior hippocampus at the level of the mammillary body, 2. the middle and superior temporal gyri and 3. the parastriate and striate cortices. Stages B0 and B1 were combined into a B0-1 group since these stages of neurofibrillary degeneration exhibit minimal involvement of the hippocampal formation. Eight of our 39 cases (25 %) demonstrated no inferior temporal or middle frontal Aβ immunoreactive plaques and were comprised of 7 cases with stage B1 and 1 case with stage B2 neurofibrillary degeneration, compatible with possible primary age-related tauopathy (PART) [7]. All other cases with neocortical tauopathy demonstrated Aβ plaques and neuritic plaques in line with AD neuropathologic changes. There was no evidence of neocortical astrocytic plaques, tufted astrocytes, argyrophillic grains, oligodendroglial coiled bodies, Pick bodies or any other features of non-AD neuronal tauopathy in any these cases.

**Table 1:** Alzheimer disease neuropathologic staging of 39 autopsy cases

Case #	Age	Gender	Amyloid	p-MAPT (NIA/AA)	p-MAPT (Braak)	CERAD	NIA-AA Category
1	68	F	A0	B0	0	C0	None (possible PART)
2	76	F	A1	B0	0	C0	Low
3	65	F	A1	B0	0	C0	Low
4	82	M	A2	B1	I	C1	Low
5	71	M	A0	B1	I	C0	None (possible PART)
6	67	M	A0	B1	I	C0	None (possible PART)
7	65	F	A0	B1	I	C0	None (possible PART)
8	82	F	A0	B1	II	C0	None (possible PART)
9	75	M	A0	B1	II	C0	None (possible PART)
10	74	M	A0	B1	II	C0	None (possible PART)
11	68	F	A0	B1	I	C0	None (possible PART)
12	83	F	A1	B1	II	C2	Low
13	84	F	A1	B1	II	C0	Low
14	87	M	A1	B1	II	C1	Low
15	75	F	A1	B1	II	C1	Low
16	79	M	A1	B1	II	C1	Low
17	77	F	A2	B1	II	C1	Low
18	73	M	A2	B1	II	C2	Low
19	76	M	A2	B1	II	C2	Low
20	76	M	A2	B1	II	C2	Low
21	68	M	A3	B1	II	C1	Low
22	71	M	A3	B1	II	C1	Low
23	83	M	A0	B2	III	C0	None (possible PART)
24	83	F	A1	B2	III	C0	Low
25	81	M	A1	B2	III	C1	Low
26	80	M	A2	B2	III	C1	Intermediate
27	77	F	A2	B2	III	C1	Intermediate
28	75	F	A2	B2	IV	C2	Intermediate
29	84	F	A2	B2	IV	C2	Intermediate
30	83	M	A2	B2	III	C2	Intermediate
31	84	F	A3	B2	III	C2	Intermediate
32	70	F	A2	B3	VI	C3	Intermediate
33	91	M	A3	B3	V	C1	Intermediate
34	89	M	A3	B3	V	C2	High
35	80	F	A3	B3	VI	C3	High
36	93	F	A3	B3	VI	C3	High
37	63	M	A3	B3	VI	C3	High
38	71	M	A3	B3	V	C3	High
39	85	M	A3	B3	V	C3	High

**Fig. 1:**
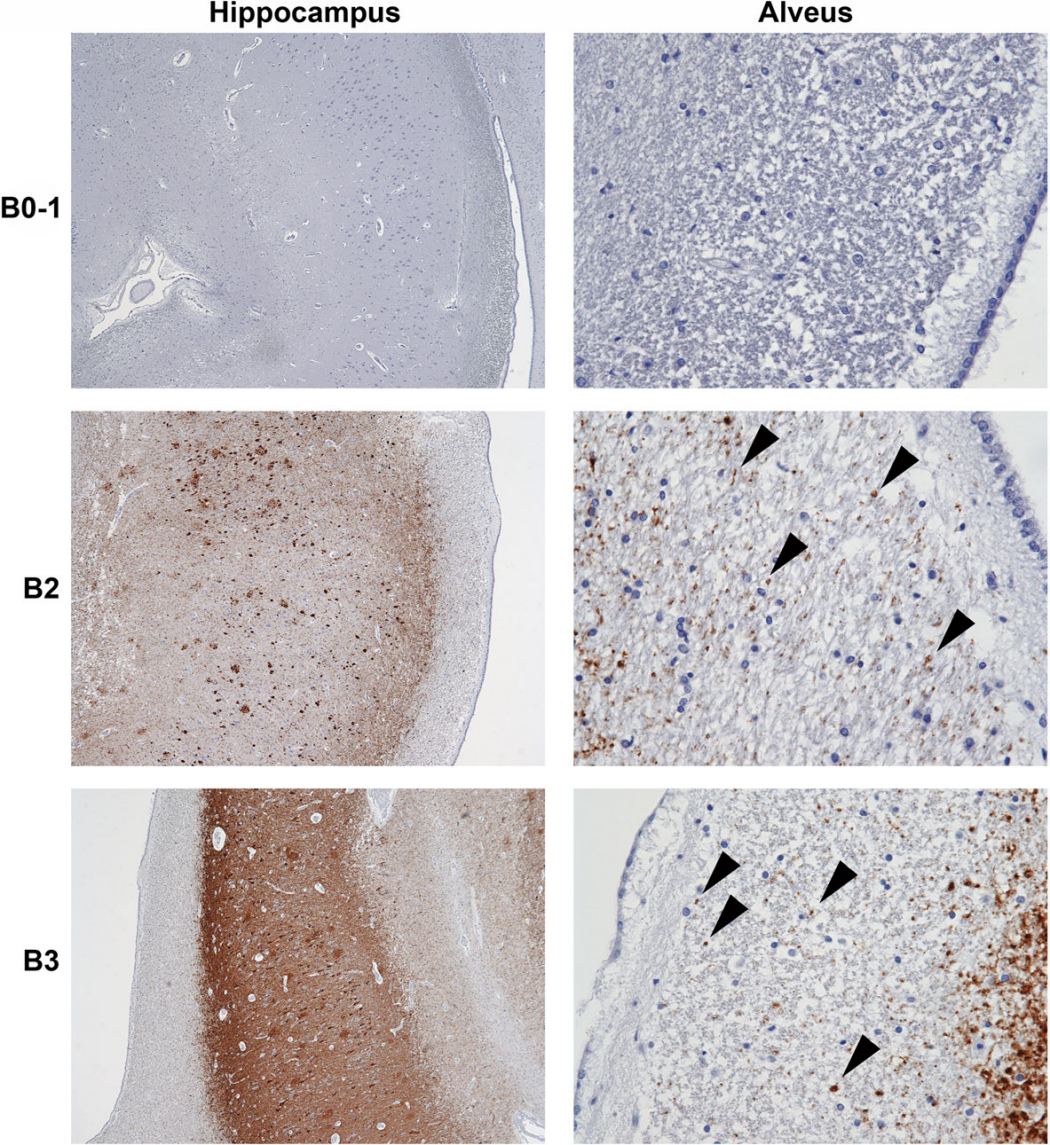
Phospho-MAPT neuropathology is present in the alveus in cases with hippocampal neurofibrillary degeneration. Left panels (original magnifications 10x): AT8 immunostaining in the hippocampal CA1 sector is demonstrated in representative cases with NIA/AA stages B0-1 (top-left), B2 (middle-left) or B3 (bottom-left) neurofibrillary degeneration. Right panels (original magnifications 200x): A case with B0-1 neurofibrillary degeneration demonstrated no axonal staining in the alveus (upper-right panel). Cases with B2 and B3 neurofibrillary degeneration (middle-right and lower-right panels, respectively) demonstrated punctate and wavy axonal AT8 staining in the alveus (arrowheads)

**Fig. 2:**
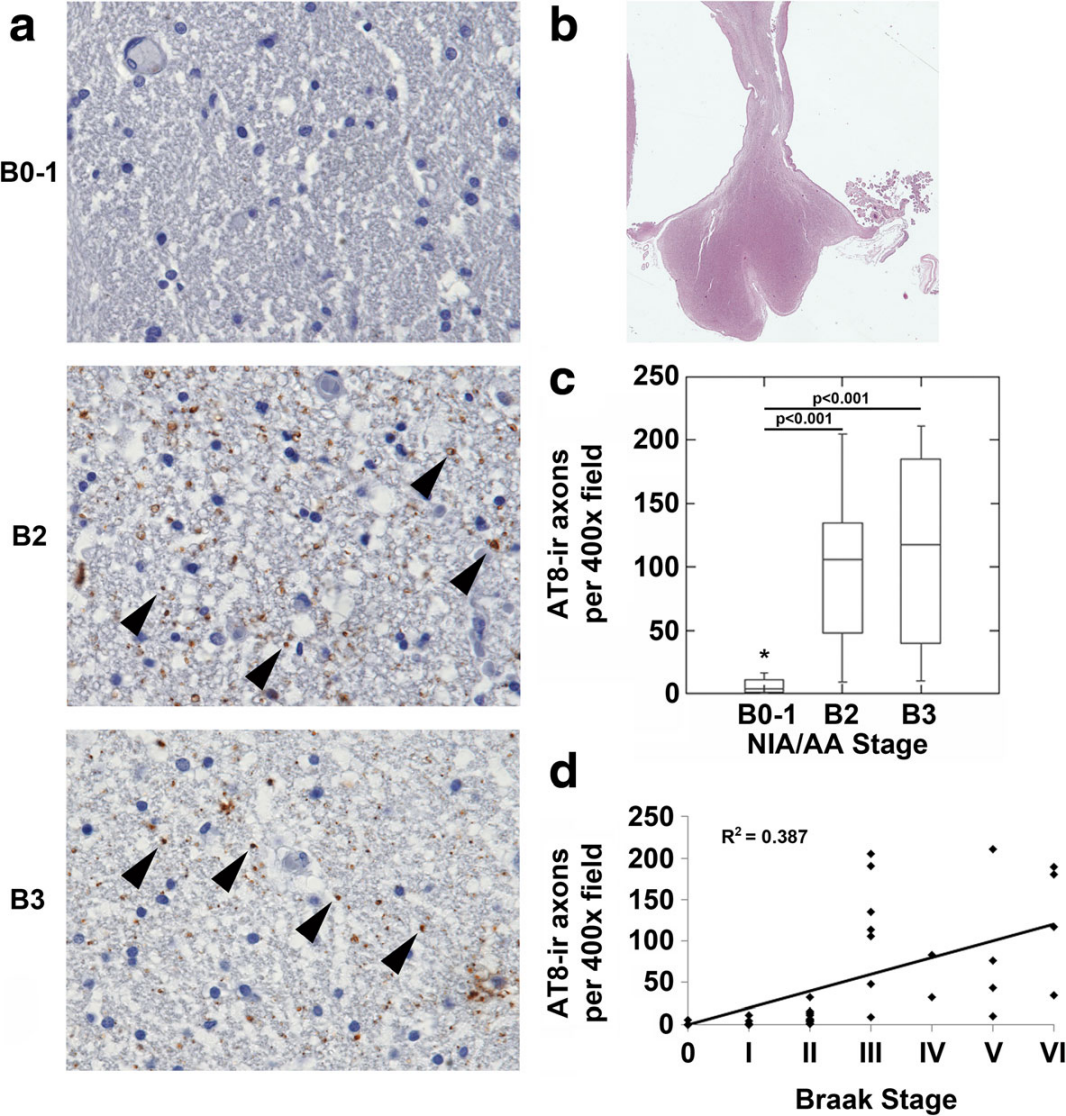
The body of the fornix demonstrates p-MAPT immunoreactive axons in NIA/AA stages B2 and B3. **a**. Images (original magnifications 400x) from coronal sections of the body of the fornix from representative cases with NIA/AA stages B0-1, B2 or B3 neurofibrillary degeneration are shown. Cases with B0-1 neurofibrillary degeneration demonstrated no punctate axonal staining in the fornix (upper left panel). Cases with B2 and B3 neurofibrillary degeneration (middle-left and lower-left panels, respectively) demonstrated punctate axonal AT8 staining in the fornix (arrowheads). **b**. Representative macroscopic image (original magnification 10x) of and H&E stained section of the body of the fornix. **c**. Cases with B2 and B3 neurofibrillary degeneration demonstrated increased AT8-immunoreactive (AT8-ir) axons compared to cases with B0-1 neurofibrillary degeneration (p <0.001, ANOVA; Bonferroni post-hoc test comparisons as indicated in the figure panel). **d**. Correlation analysis revealed a significant correlation between Braak stages of neurofibrillary degeneration and AT8-ir axons in the fornix (*n* = 39, *R* = 0.622, *p* < 0.001)

In AT8-immunostained sections of the anterior hippocampus, punctate and wavy AT8 immunoreactive (AT8-ir) processes in the alveus, a major efferent tract of the hippocampal formation, were conspicuous in cases with NIA/AA stages B2 and B3, but not in a case with NIA/AA stage B0-1, neurofibrillary degeneration (Fig. 1). We examined AT8 immunoreactivity in coronal sections of the body of the fornix and discovered small, round, dot-like profiles of AT8-ir axonal staining only in NIA/AA stages B2 and B3 of neurofibrillary degeneration (Fig. 2a). Image analysis demonstrated that the fornix showed exceedingly rare AT8-ir puncta in NIA/AA stages B0-1, but demonstrated frequent AT8-ir puncta in NIA/AA stage B2 (Fig. 2a and c). We observed no further increase in AT8-ir axon profiles in the fornix in NIA/AA stage B3 (Fig. 2c). Correlation analysis demonstrated a significant correlation between the Braak stage of neurofibrillary degeneration and the density of AT8-ir axons in the fornix (Fig. 2d).

**Fig. 3:**
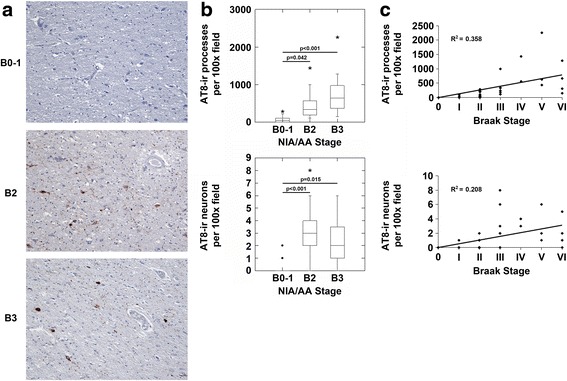
The septum demonstrates p-MAPT neuropathology in NIA/AA stages B2 and B3. **a**. Images (original magnification 100x) from sections of the septum from representative cases with NIA/AA stages B0-1, B2 or B3 neuropathology are shown. Cases with B0-1 neurofibrillary degeneration demonstrated no significant process or neuronal staining in the septum (upper panel). Cases with B2 and B3 neurofibrillary degeneration (middle and lower panels, respectively) demonstrated AT8-ir processes and neurons. **b**. Cases with B2 and B3 neurofibrillary degeneration demonstrated increased AT8-ir processes (upper graph; *p* < 0.001, ANOVA) and neurons (lower graph; *p* < 0.001, ANOVA) compared to cases with B0-1 neurofibrillary degeneration (Bonferroni post-hoc test comparisons as indicated in the figure panel). **c**. Correlation analysis revealed a significant correlation between Braak stages of neurofibrillary degeneration and both AT8-ir processes (*n* = 37, *R* = 0.599, *p* < 0.001) and AT8-ir neurons (*n* = 37, *R* = 0.456, *p* = 0.015) in the septum

**Fig. 4:**
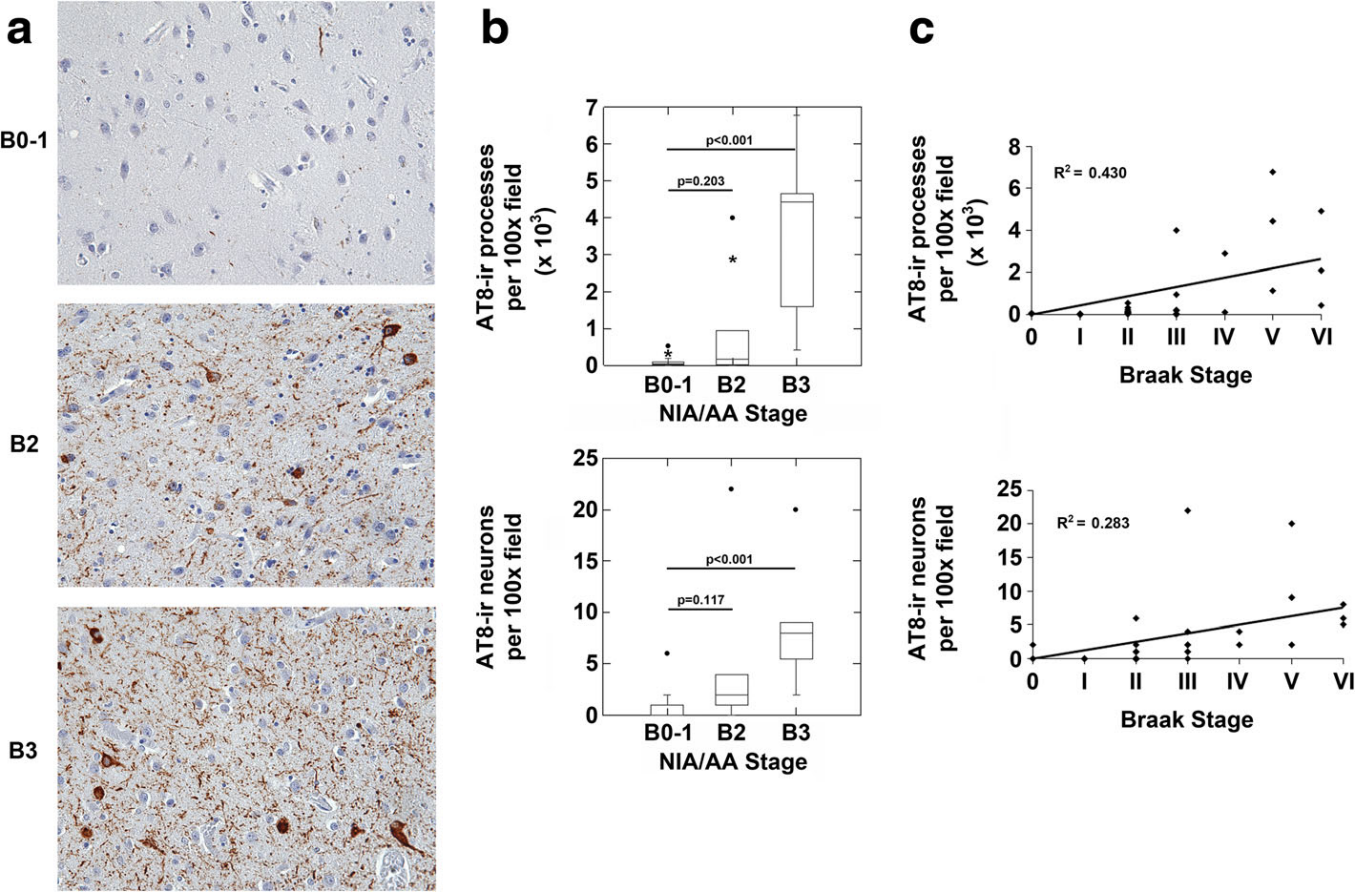
The nucleus accumbens demonstrates p-MAPT neuropathology in NIA/AA stages B2 and B3. **a**. Images (original magnification 200x) from sections of the nucleus accumbens from representative cases with NIA/AA stages B0-1, B2 or B3 neuropathology are shown. Cases with B0-1 neurofibrillary degeneration demonstrated rare AT8-ir process but no significant neuronal staining in the nucleus accumbens (upper panel). Cases with B2 and B3 neurofibrillary degeneration (middle and lower panels, respectively) demonstrated AT8-ir processes and neurons. **b**. Cases with B3 neurofibrillary degeneration demonstrated increased AT8-ir processes (upper graph; *p* < 0.001, ANOVA; Bonferroni post-hoc test comparisons as indicated) and neurons (lower graph; *p* < 0.001, ANOVA; Bonferroni post-hoc test comparisons as indicated) compared to cases with B0-1 neurofibrillary degeneration. Cases with B2 neurofibrillary degeneration demonstrated trends towards increased AT8-ir processes and neurons compared to cases with B0-1 neurofibrillary degeneration (Bonferroni post-hoc test comparisons as indicated in graph panels). **c**. Correlation analysis revealed a significant correlation between Braak stages of neurofibrillary degeneration and both AT8-ir processes (*n* = 37, *R* = 0.656, *p* < 0.001) and AT8-ir neurons (*n* = 37, *R* = 0.532, *p* = 0.002) in the nucleus accumbens

We next examined AT8 immunoreactivity in precommissural efferent target nuclei of the fornix - the septum and nucleus accumbens (NA). We detected no significant p-MAPT neuropathology in the septum in NIA/AA stages B0 and B1 (Fig. 3a and b). However, in NIA/AA stage B2 and B3, there were significant increases in AT8-ir processes and neurons in the septum (Fig. 3a and b). Correlation analysis demonstrated a significant correlation between the Braak stage of neurofibrillary degeneration and the density of AT8-ir pathology in the septum (Fig. 3c). Only rare AT8-ir neurons demonstrated granular pre-tangle staining in the nucleus accumbens(NA) in NIA/AA stages B0-1 (Fig. 4a and b). AT8-ir processes and neurons were present in the NA in NIA/AA stages B2 and B3, however they reached statistical significance only in stage B3 in our cohort (Fig. 4a and b). Correlation analysis demonstrated a significant correlation between the Braak stage of neurofibrillary degeneration and the density of AT8-ir pathology in the NA (Fig. 4c).

**Fig. 5:**
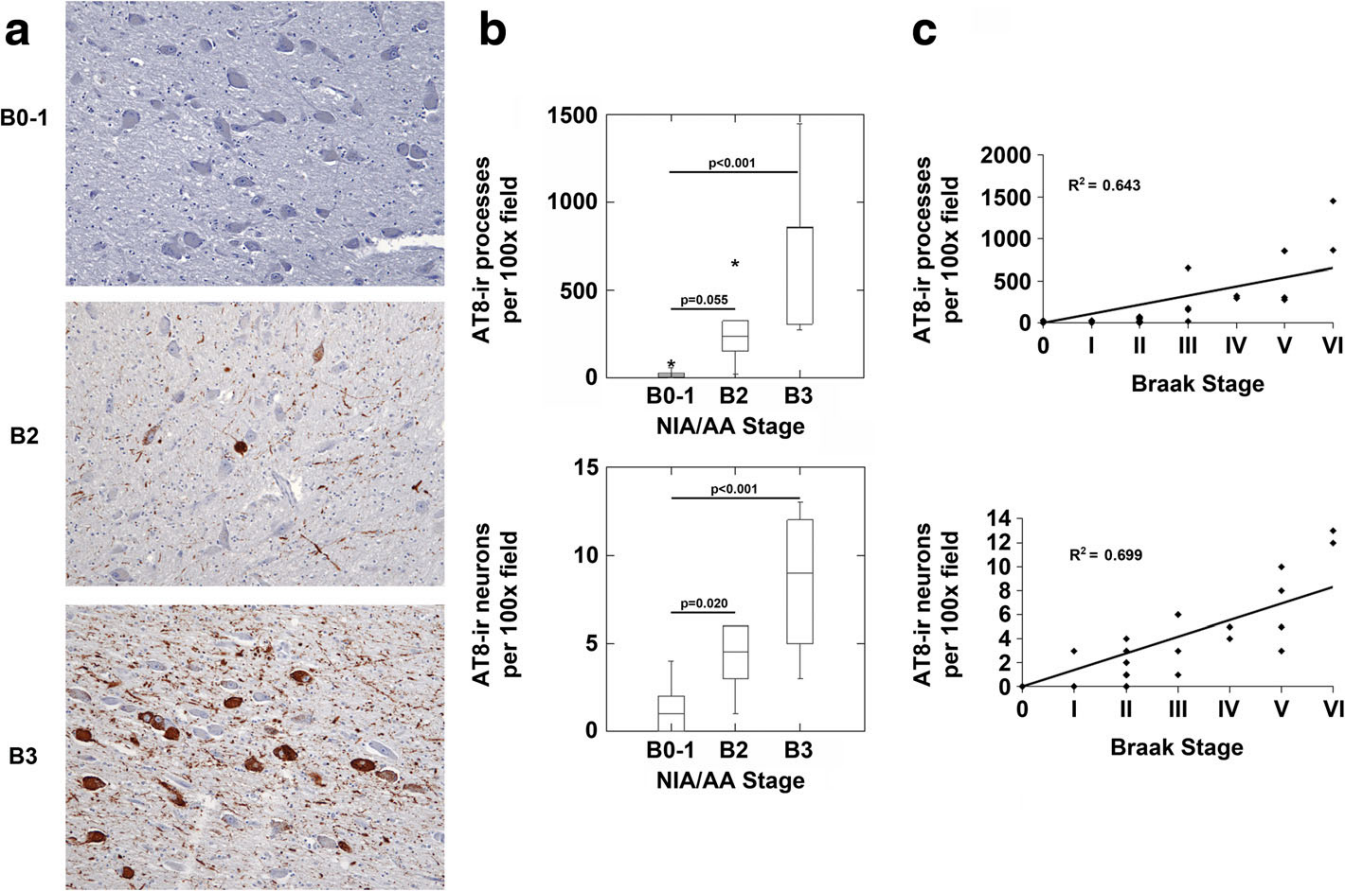
The nucleus basalis of Meynert demonstrates p-MAPT neuropathology in NIA/AA stages B2 and B3. **a**. Images (original magnification 100x) from sections of the nucleus basalis of Meynert from representative cases with NIA/AA stages B0-1, B2 or B3 neuropathology are shown. Cases with B0-1 neurofibrillary degeneration demonstrated no significant process or magnocellular neuronal staining in the nucleus basalis (top panel). Cases with B2 and B3 neurofibrillary degeneration (middle and bottom panels, respectively) demonstrated AT8-ir processes and neurons. **b**. Cases with B3 neurofibrillary degeneration demonstrated increased AT8-ir processes (upper graph; *p* < 0.001, ANOVA; Bonferroni post-hoc test comparisons as indicated) and neurons (lower graph; *p* < 0.001, ANOVA; Bonferroni post-hoc test comparisons as indicated) compared to cases with B0-1 neurofibrillary degeneration. Cases with B2 neurofibrillary degeneration demonstrated increased AT8-ir neurons and a strong trend towards increased AT8-ir processes compared to cases with B0-1 neurofibrillary degeneration (Bonferroni post-hoc test comparisons as indicated in graph panels). **c**. Correlation analysis revealed a significant correlation between Braak stages of neurofibrillary degeneration and both AT8-ir processes (*n* = 30, *R* = 0.802, *p* < 0.001) and AT8-ir neurons (*n* = 30, R = 0.836, *p* < 0.001) in the nucleus basalis of Meynert

In the nucleus basalis of Meynert, magnocellular neurons with p-MAPT neuropathology in NIA/AA stages B0 and B1 were rare (Fig. 5a and b) but there were significant increases in AT8-ir processes and neurons in NIA/AA stages B2 and B3 (Fig. 5a and b). Correlation analysis demonstrated strong correlation between the Braak stage of neurofibrillary degeneration and the density of AT8-ir pathology in the nucleus basalis of Meynert (Fig. 5c).

**Fig. 6:**
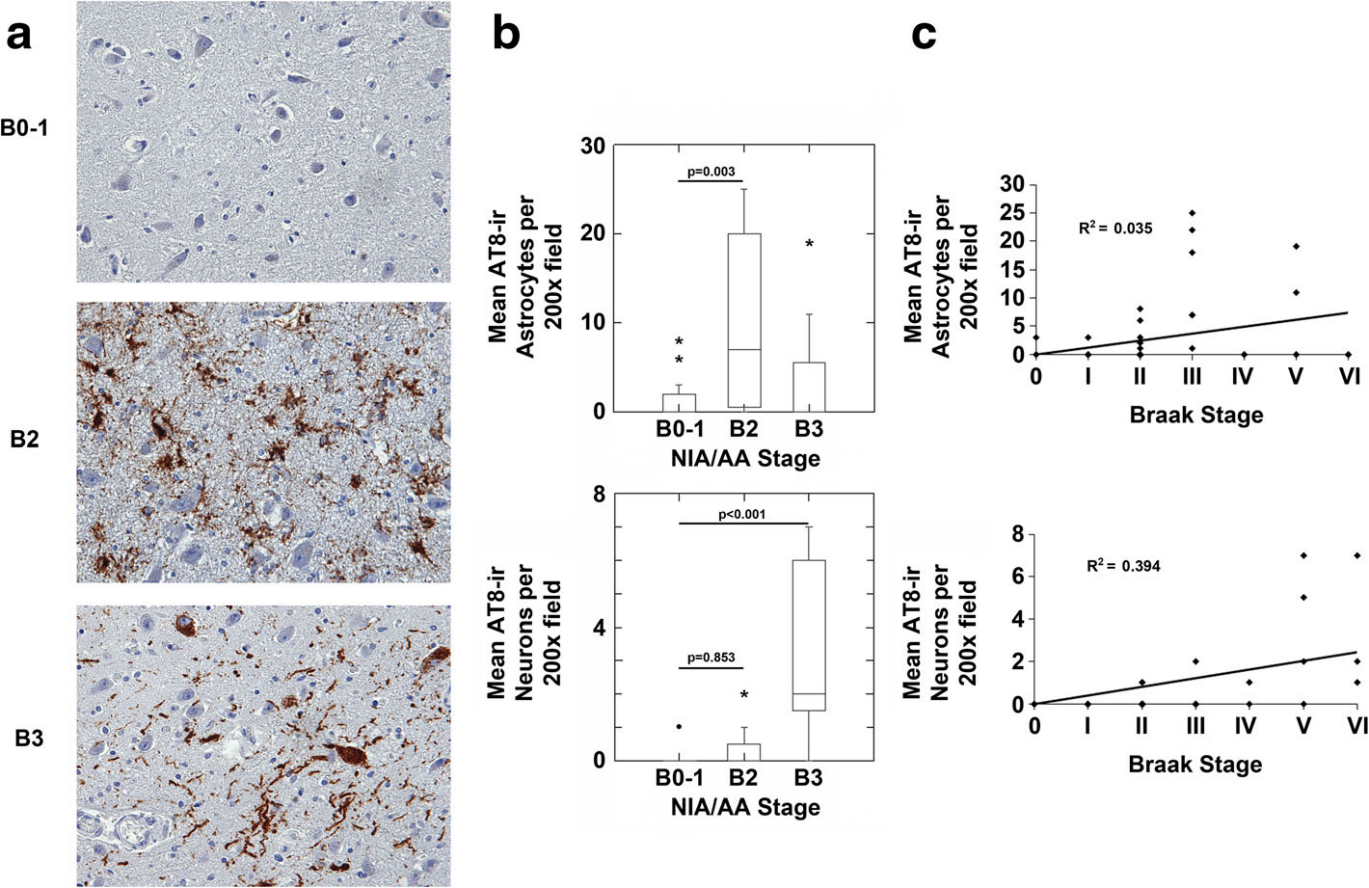
The mammillary bodies demonstrated AT8-ir astrocytes most prominently in NIA/AA stage B2 and AT8-ir neurons in NIA/AA stage B3. **a**. Images (original magnification 200x) from sections of the mammillary bodies from representative cases with NIA/AA stages B0-1, B2 or B3 neuropathology are shown. Cases with B0-1 neurofibrillary degeneration demonstrated no significant neuronal AT8 staining in the nucleus basalis (upper panel). Cases with B2 neurofibrillary degeneration (middle panel) demonstrated predominantly AT8-ir astrocytes whereas cases with B3 neurofibrillary degeneration (lower panel) demonstrated predominantly AT8-ir neurons. **b**. Cases with B2 neurofibrillary degeneration demonstrated increased AT8-ir astrocytes (upper graph; *p* < 0.004, ANOVA; Bonferroni post-hoc test comparisons as indicated) but no significant increase in AT8-ir neurons. Cases with B3 neurofibrillary degeneration demonstrated increased AT8-ir neurons compared to B0-1 and B2 (lower graph; *p* < 0.001, ANOVA; Bonferroni post-hoc test comparisons as indicated). **c**. Correlation analysis revealed a significant correlation between Braak stages of neurofibrillary degeneration and AT8-ir neurons (*n* = 37, *R* = 0.628, *p* < 0.001). There was no significant correlation between Braak stage and AT8-ir astrocytes (*n* = 37, *R* = 0.188, *p* = 1) in the mammillary bodies

Sections of the mammillary bodies (MB) were remarkable for a mix of neuronal and astroglial tauopathy (Fig. 6). In NIA/AA stages B0-1, only very rare AT8-ir astrocytes and AT8-ir neurons were seen in the MB (Fig. 6a and b). The density of AT8-ir astrocytes, but not AT8-ir neurons, was markedly increased in the MB in NIA/AA stage B2. The thorn-shaped morphology of the p-MAPT immunoreactive astrocytes was consistent with aging-related tau astrogliopathy (ARTAG) [9]. In NIA/AA stage B3, there was a significant increase in AT8-ir neurons as well as admixed AT8-ir astrocytes in the MB (Fig. 6a and b). Correlation analysis demonstrated no relationship between the Braak stage of neurofibrillary degeneration and the density of AT8-ir astrocytes in the MB (Fig. 6c, top panel) but there was a significant correlation between the Braak stage of neurofibrillary degeneration and the density of AT8-ir neurons in the MB (Fig. 6c, bottom panel). Correlation analysis also demonstrated a significant correlation between increasing age and increased AT8-ir astrocytes in the MB (*n* = 37; *R* = 0.473, *p* < 0.004) but there was no significant difference in the density of AT8-ir astrocytes in the mammillary bodies between genders (Male: 5 ± 2 AT8-ir astrocytes vs Female: 2 ± 2 AT8-ir astrocytes; *p* = 0.285, unpaired Student’s *t* test).

**Fig. 7:**
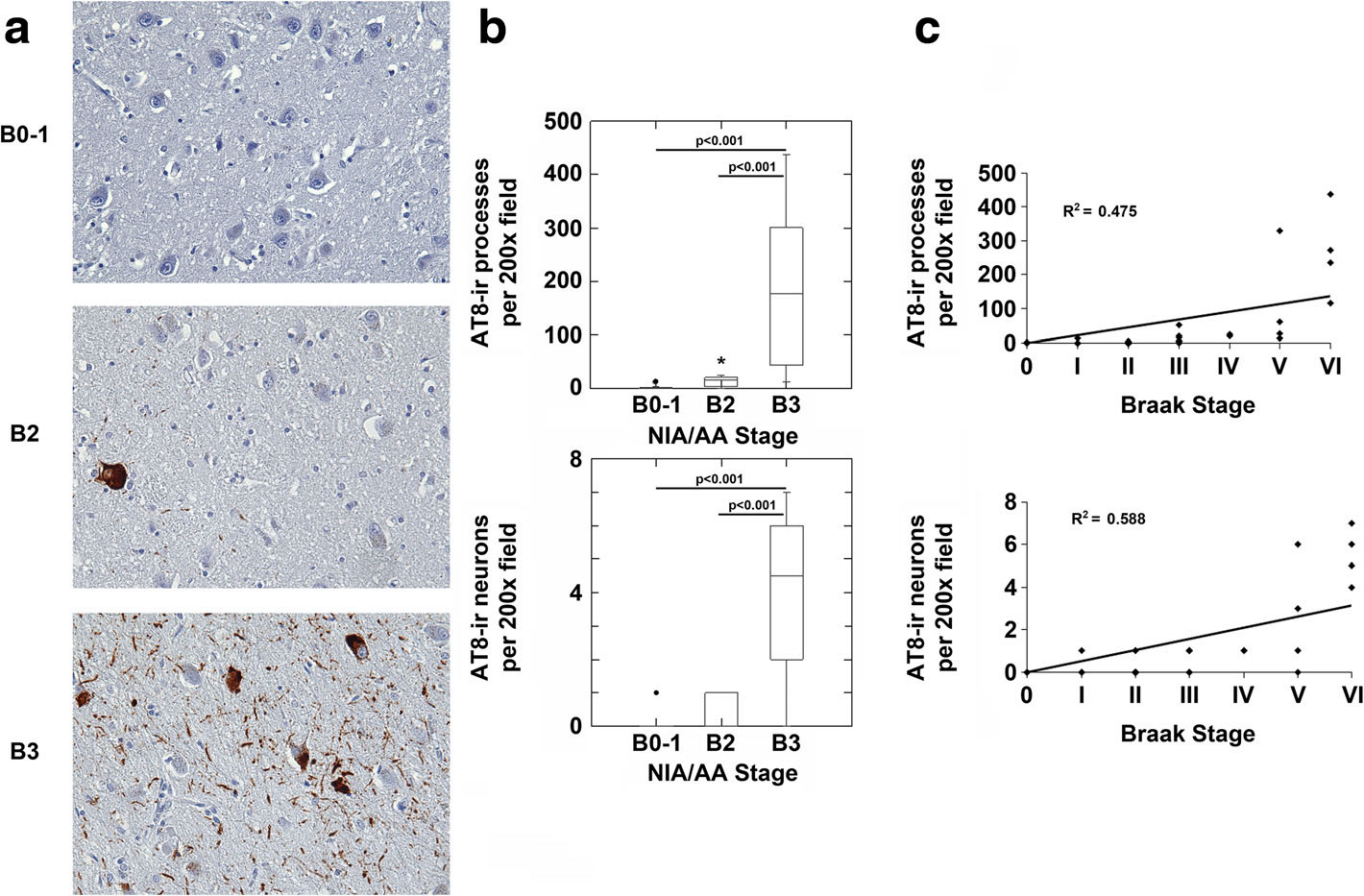
The anterior thalamic nucleus demonstrates p-MAPT neuropathology predominantly in NIA/AA stage B3. **a**. Images (original magnification 200x) from sections of the anterior thalamic nucleus from representative cases with NIA/AA stages B0-1, B2 or B3 neuropathology are shown. Cases with B0-1 neurofibrillary degeneration demonstrated no significant process or neuronal staining in the anterior thalamic nucleus (upper panel). Cases with B2 neurofibrillary degeneration (middle panel) demonstrated only very rare AT8-ir processes and neurons. Cases with B3 neurofibrillary degeneration (bottom panel) demonstrated frequent AT8-ir processes and neurons. **b**. Cases with B3 neurofibrillary degeneration, but not B2 neurofibrillary degeneration, demonstrated increased AT8-ir processes (upper graph; *p* < 0.001, ANOVA; Bonferroni post-hoc test comparisons as indicated) and neurons (lower graph; *p* < 0.001, ANOVA; Bonferroni post-hoc test comparisons as indicated) compared to cases with B0-1 neurofibrillary degeneration. **c**. Correlation analysis revealed a significant correlation between Braak stages of neurofibrillary degeneration and both AT8-ir processes (*n* = 37, *R* = 0.689, *p* < 0.001) and AT8-ir neurons (*n* = 37, *R* = 0.767, *p* < 0.001) in the anterior thalamic nucleus

AT8 immunostains performed on sections of the anterior thalamic nucleus (ATN) (Fig. 7) revealed no significant AT8-ir processes and neurons in the ATN in NIA/AA stages B0-1 or NIA/AA stage B2 (Fig. 7a and b). Significant increases in AT8-ir processes and neurons in the ATN were seen only in NIA/AA stage B3 (Fig. 7a and b). Correlation analysis demonstrated a significant correlation between the Braak stage of neurofibrillary degeneration and the density of AT8-ir pathology in the ATN (Fig. 7c).

**Fig. 8:**
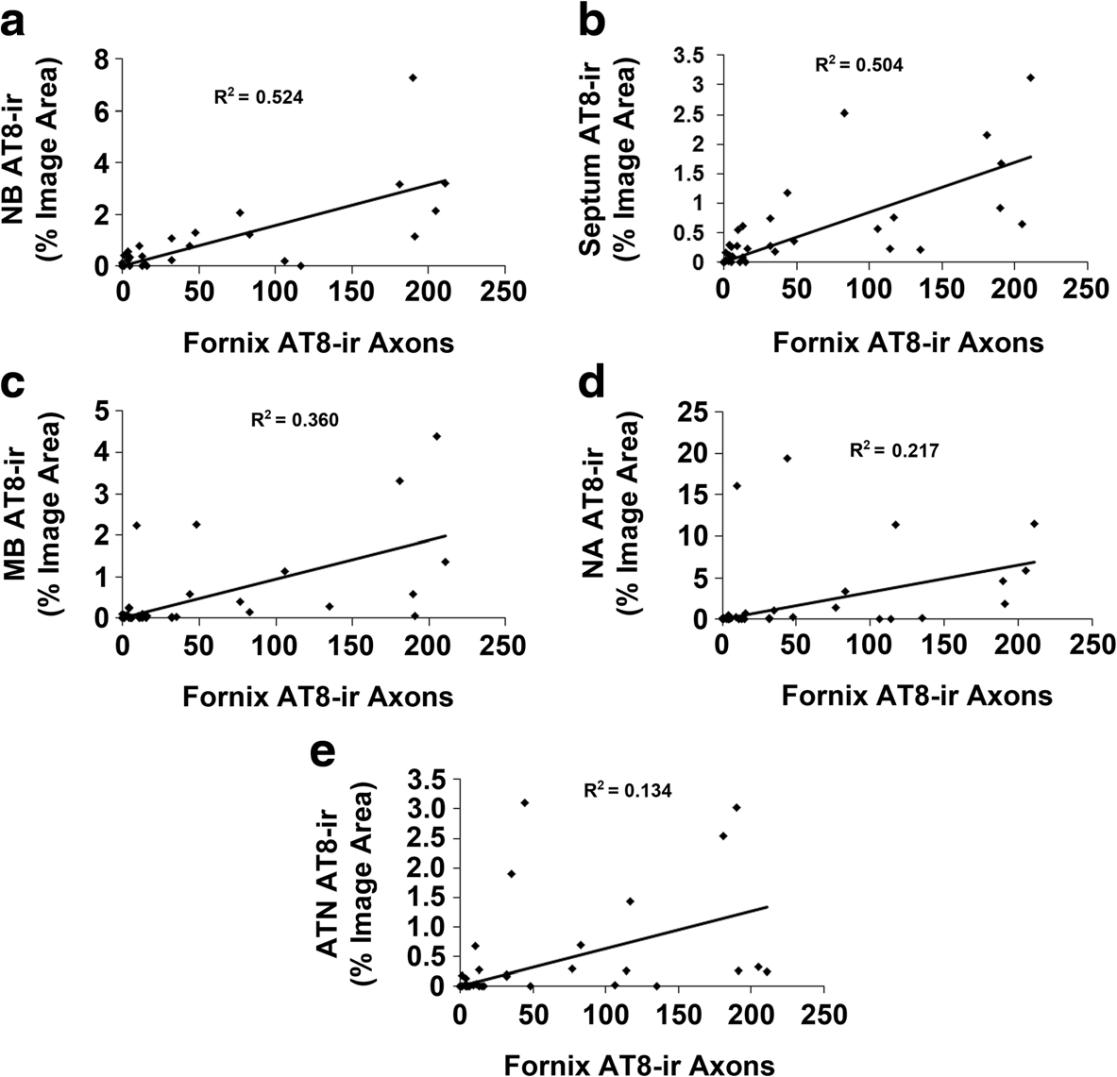
Correlation analysis of p-MAPT neuropathology in fornix and basal forebrain nuclei. Correlation analysis (Pearson Correlation Coefficient with Bonferroni probabilities) revealed a significant correlation between AT8-ir axons in the fornix and p-MAPT neuropathology in the nucleus basalis of Meynert (**a**; *n* = 30, *R* = 0.724, *p* < 0.002), septum (**b**; *n* = 36, *R* = 0.710, *p* < 0.003) and mammillary bodies (**c**; *n* = 37, *R* = 0.600, *p* < 0.03). No significant correlation was present between AT8-ir axons in the fornix and p-MAPT neuropathology in the nucleus accumbens (**d**; *n* = 37, *R* = 0.466, *p* = 0.328) or in the anterior thalamic nucleus (**e**; *n* = 37, *R* = 0.366, *p* = 1)

We performed correlation analyses to examine the relationship of AT8-ir axons in the fornix with AT8-immunoreactivity in the basal forebrain nuclei (Fig. 8). There was a significant correlation between p-MAPT neuropathology in the fornix and p-MAPT neuropathology in the nucleus basalis of Meynert (Fig. 8a) and the septum (Fig. 8b). In the mammillary body, there was a significant correlation between p-MAPT neuropathology in the fornix and overall neuronal and astroglial p-MAPT neuropathology (Fig. 8c), however, there was no significant correlation with neuronal tauopathy alone (*n* = 37; *R* = 0.327, *p* = 0.288) and only a trend towards correlation with astroglial tauopathy alone (*n* = 37; *R* = 0.421, *p* = 0.057) in our cohort. The correlations between p-MAPT neuropathology in the fornix and p-MAPT neuropathology in the NA (Fig. 8d) and in the ATN (Fig. 8e) were not statistically significant.

## Discussion

In this autopsy immunohistology study, we hypothesized that the fornix, a major efferent outflow tract of the hippocampal formation, is a conduit for the propagation of p-MAPT neuropathology from the hippocampus to the basal forebrain in early AD. To test this hypothesis, we analyzed p-MAPT immunostaining in the fornix and its efferent targets in the basal forebrain in human brain autopsies with early to advanced AD neuropathologic changes. We expected that the fornix would recapitulate a core feature of the Braak and NIA/AA staging systems in the spread of p-MAPT neuropathology across connected brain regions with involvement downstream of the hippocampal formation. Indeed, in cases of B0-1 neurofibrillary degeneration with none or minimal involvement of the hippocampal formation by p-MAPT neuropathology, we observed no significant p-MAPT (AT8) immunostaining in the fornix or efferent target nuclei. In cases of B2 neurofibrillary degeneration, we observed p-MAPT staining in axonal processes of the fornix and in precommissural nuclei (septum and nucleus accumbens). The mammillary body, innervated by postcommissural fibers of the fornix, demonstrated tauopathy that was predominantly astroglial in stage B2 and that was mixed neuronal and astroglial in stage B3. P-MAPT neuropathology was sparse in the ATN, an efferent target of the fornix and of the mammillary bodies via the mammillothalamic tract, in NIA/AA stage B2 but rose significantly in NIA/AA stage B3. These results of our autopsy study demonstrate that the fornix is a conduit for p-MAPT neuropathology in AD and are compatible with p-MAPT neuropathology propagation from the hippocampal formation to the basal forebrain nuclei via the fornix as AD-related tauopathy progresses.

Braak and Braak [2–4] described the stereotypical propagation of p-MAPT neuropathology in AD which is the basis of the neurofibrillary degeneration staging system that bears their name. The serial propagation between synaptically connected regions, like the entorhinal cortex and the hippocampal formation, supports the hypothesis that axons and synapses play an important role. Mechanistically, recent work has supported the concept that MAPT neuropathology is propagated along connected brain regions and that the synapse itself is an important factor for, and perhaps a substrate of, transneuronal p-MAPT neuropathology propagation [5, 6, 8]. Our findings, while generally not surprising in the context of these lines of evidence, are important because they fill a gap in our knowledge of p-MAPT propagation in structures not routinely sampled in AD neuropathologic examinations.

Since cognitive impairment in AD is correlated with the spread of pathologic tau [14, 16], it is possible that the risk of progression from MCI to AD might be predicted by the spread of p-MAPT from the hippocampus via the fornix. Consistent with this premise, elevated CSF MAPT and p-MAPT have become important biomarkers in the clinical diagnosis of AD [19]. Diffusion tensor imaging MRI studies have suggested that increased diffusion measures in the fornix may predict progression in AD and are suggested to relate to p-MAPT related axonal injury [1, 18], however, definitive pathologic correlation is lacking. Ongoing work in *in vivo* MAPT imaging [20] might also facilitate detection of p-MAPT propagation in the fornix.

We performed correlation analyses of p-MAPT staining in the fornix and basal forebrain nuclei to further probe the tauopathy patterns in these interconnected structures. The density of p-MAPT staining in the fornix and all the basal forebrain structures demonstrated significant correlation with the Braak stage of neurofibrillary degeneration, compatible with the progressive spread of tauopathy to these structures as AD neurofibrillary degeneration advances along the regional pattern described by Braak and Braak [2–4]. Interestingly, correlation analysis of the density of fornix tauopathy and the density of tauopathy in basal forebrain structures was not uniform. The strongest correlation between tauopathy in the fornix and nucleus basalis of Meynert suggests the possibility that nucleus basalis projections to the hippocampus through the fornix contribute to fornix tauopathy in addition to efferent hippocampal formation projections. Tauopathy in the fornix was also significantly correlated with tauopathy in the septum but was not significantly correlated with tauopathy in the NA or ATN. In our cohort, there were cases with relatively higher levels of tauopathy in the NA and ATN but lower levels of fornix p-MAPT that contributed to this lack of correlation. Since these cases represent NIA/AA staged B2 and B3, lack of concordance might be due to spread of p-MAPT neuropathology to the basal forebrain via other pathways, for example via the amygdala in the case of the NA, or could reflect decreases in fornix p-MAPT-positive axons due to axonal loss.

Our autopsy study also highlighted two additional tauopathies that can involve the mesiotemporal lobe and basal forebrain: PART [7] and ARTAG [10]. PART is characterized by neurofibrillary degeneration in a pattern resembling AD but in the absence of significant Aβ neuropathology. Since we performed Aβ immunostains only on the middle and inferior temporal gyri and are unable to completely exclude the possibility of amyloid plaques in other neocortical regions, we categorized patients lacking amyloid plaques as possible PART. Seven brains in our study demonstrated possible PART confined to the entorhinal cortex with sparse involvement of the CA1 sector of the hippocampus (Braak stage I-II). Overall, p-MAPT staining was sparse in the fornix in cases with entorhinal neurofibrillary degeneration and there was no significant difference in fornix p-MAPT axonal staining between the 12 brains with B1 Alzheimer disease neuropathologic changes (8.7 ± 2.6 p-MAPT-positive axonal profiles) and the seven brains with possible Braak stage I-II PART (5.7 ± 2.2 p-MAPT-positive axonal profiles). Only one brain in our autopsy series demonstrated possible Braak stage III PART and it showed approximately ½ of the mean axonal p-MAPT staining for the B2 neurofibrillary degeneration group. These results suggest that fornix p-MAPT neuropathology can be seen in Braak stage ≥ III PART. Since dementia is uncommon in patients with PART [7], the finding of p-MAPT-related axonal damage in the fornix highlights the potential importance of *in vivo* Aβ imaging and biomarkers to differentiate patients with AD who are more likely to progress to dementia.

Additionally, several brains demonstrated abundant p-MAPT immunoreactive thorn-shaped astrocytes in the MB. We suspect that this finding is most likely related to ARTAG [10] as it was seen in all NIA/AA stages of neurofibrillary degeneration and its density correlated with patient age. However it is interesting to note that MB tau astrogliopathy was most prominent in NIA/AA stage B2 of neurofibrillary degeneration and preceded neuronal tau pathology in the MB in our autopsy cohort. Furthermore, there was a modest but statistically significant correlation between the combination of astroglial and neuronal tauopathy in the MB and tauopathy in the fornix. Although we favor these findings to represent ARTAG, they could indicate a role for tau astrogliopathy in tau propagation from the fornix to the mammillary body. Further studied are needed to address the significance of tau astrogliopathy in the MB.

The major limitations of our study are its cross-sectional design and its small sample size. We have inferred from the cross-sectional design of this autopsy study that the fornix propagates p-MAPT neuropathology from the hippocampus to efferent structures in the medial basal forebrain. However, *in vivo* tau imaging, DTI and post-mortem neuropathologic correlation in patients with neuropsychiatric evaluations will be needed to determine if this is the true progression sequence of AD-related tau pathology from the hippocampus and its significance with respect to the risk of progression from MCI to dementia. Out of concern for the possible impairment of antigen immunostaining with chronic formalin-fixation, we limited our study to archived autopsies no older than 2 years. Furthermore, as p-MAPT neuropathology can be associated with diverse brain diseases, we eliminated cases with brain pathologies that might affect the pattern of p-MAPT neuropathology or that might affect our immunohistochemical detection of p-MAPT neuropathology. Despite the resultant small sample size or our study, our data robustly demonstrate that axonal AT8 immunoreactivity in the fornix is present in the limbic stage of AD neuropathology and does not precede hippocampal involvement. It is possible that tau antibodies that detect earlier phosphorylation states or oligomeric tau could render additional insights into tau propagation among the medial temporal lobe and basal forebrain. Nonetheless, our findings largely support the expected model of AD-related p-MAPT neuropathology propagation from the hippocampus to the basal forebrain via the fornix in NIA/AA stages B2 and B3 of neurofibrillary degeneration. However, our findings are compatible with the possibilities that the fornix carries p-MAPT to the hippocampal formation from the nucleus basalis of Meynert and that basal forebrain nuclei may propagate p-MAPT neuropathology from structures other than the fornix.

## Conclusions

In conclusion, as radiologic modalities that detect MAPT neuropathology and MAPT-related axonal damage continue to show promise for the *in vivo* diagnosis and prognostication for patients with early AD, it is critical that we have a full understanding of MAPT neuropathology propagation patterns in the aging human brain. Our study confirms that the fornix demonstrates p-MAPT neuropathology in NIA/AA stages B2 and B3 of neurofibrillary degeneration and suggests that the fornix propagates p-MAPT neuropathology from the hippocampal formation to the basal forebrain as AD progresses. Additional work is necessary to further understand p-MAPT neuropathology propagation in these interconnected structures and to correlate MRI changes and cognitive data with p-MAPT neuropathology in these structures in AD.

## Abbreviations

AD: Alzheimer disease
ANOVA: Analysis of variance
ARTAG: Aging-related tau astrogliopathy
AT8-ir: AT8-immunoreactive
ATN: Anterior thalamic nucleus
Aβ: Amyloid-beta
DTI: Diffusion tensor imaging
H&E: Hematoxylin and eosin
MAPT: Microtubule-associated protein tau
MB: Mammillary body
MCI: Mild cognitive impairment
MRI: Magnetic resonance imaging
NA: Nucleus accumbens
NIA/AA: National Institute on Aging/Alzheimer Association
PART: Primary age-related tauopathy
p-MAPT: Phospho-tau/MAPT.
